# Supplementary material for: Characterization of the Neurospora crassa Galactosaminogalactan Biosynthetic Pathway
Source: Microorganisms. 2024 Jul 23;12(8):1509. doi: 10.3390/microorganisms12081509 (PMC11356417; doi:10.3390/microorganisms12081509)
Supplement: Supplementary file 1 [file microorganisms-12-01509-s001.zip › microorganisms-3115855-supplementary.pptx]

## Slide 1
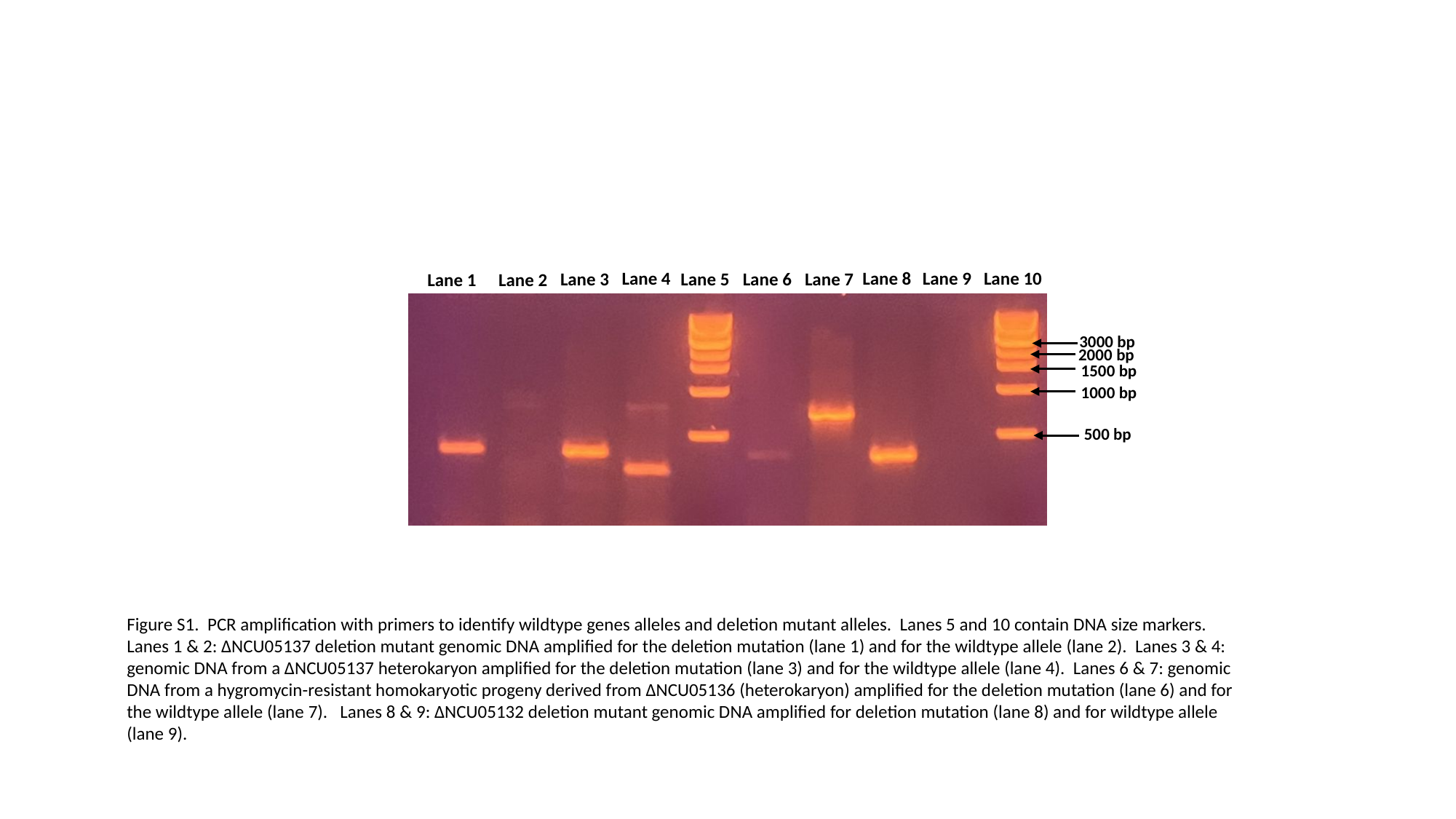

Lane 8
Lane 4
Lane 9
Lane 10
Lane 5
Lane 3
Lane 6
Lane 7
Lane 1
Lane 2
3000 bp
2000 bp
1500 bp
1000 bp
500 bp
Figure S1. PCR amplification with primers to identify wildtype genes alleles and deletion mutant alleles. Lanes 5 and 10 contain DNA size markers. Lanes 1 & 2: ∆NCU05137 deletion mutant genomic DNA amplified for the deletion mutation (lane 1) and for the wildtype allele (lane 2). Lanes 3 & 4: genomic DNA from a ∆NCU05137 heterokaryon amplified for the deletion mutation (lane 3) and for the wildtype allele (lane 4). Lanes 6 & 7: genomic DNA from a hygromycin-resistant homokaryotic progeny derived from ∆NCU05136 (heterokaryon) amplified for the deletion mutation (lane 6) and for the wildtype allele (lane 7). Lanes 8 & 9: ∆NCU05132 deletion mutant genomic DNA amplified for deletion mutation (lane 8) and for wildtype allele (lane 9).

## Slide 2
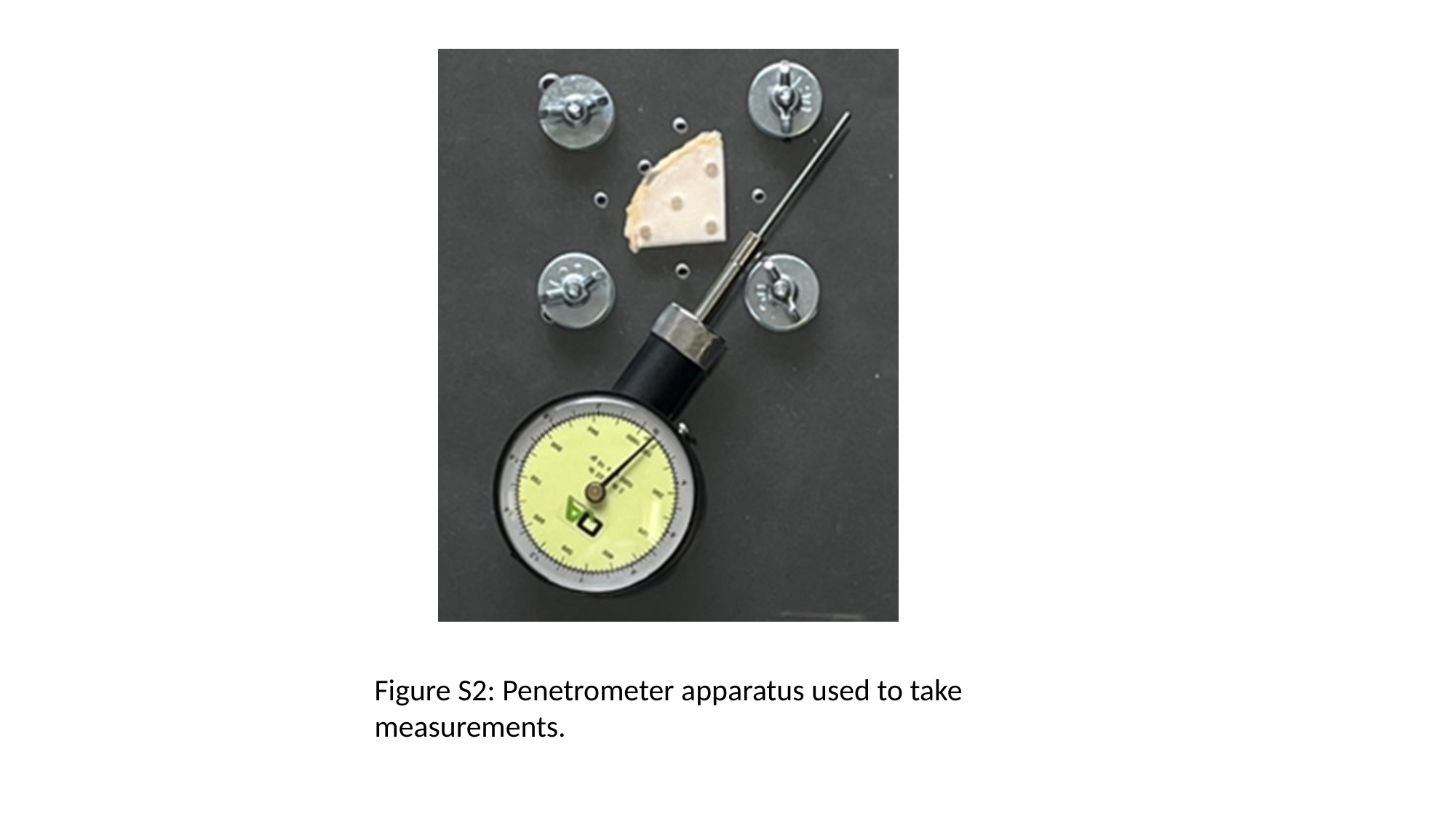

Figure S2: Penetrometer apparatus used to take measurements.

## Slide 3
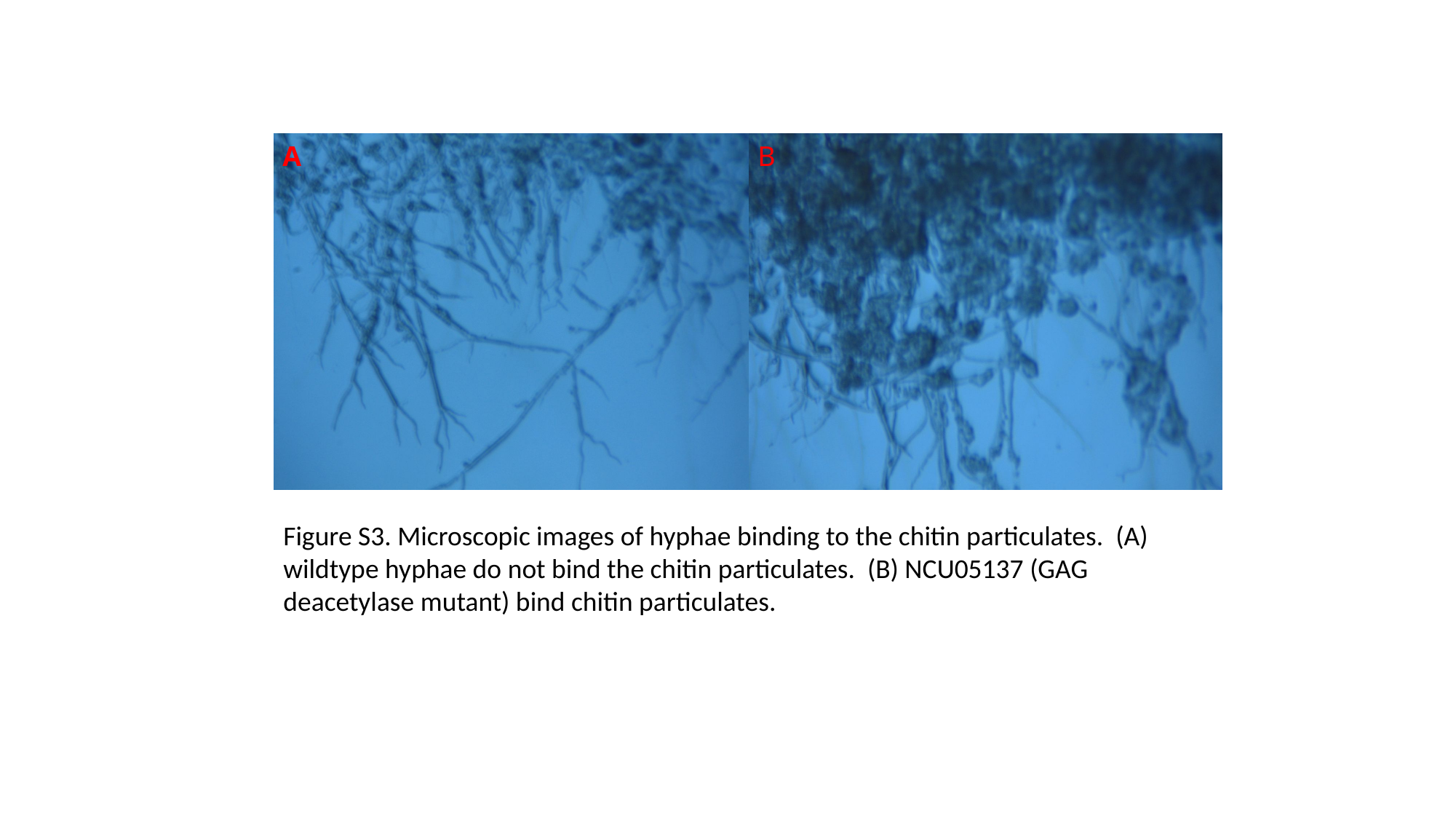

A
B

## Slide 4
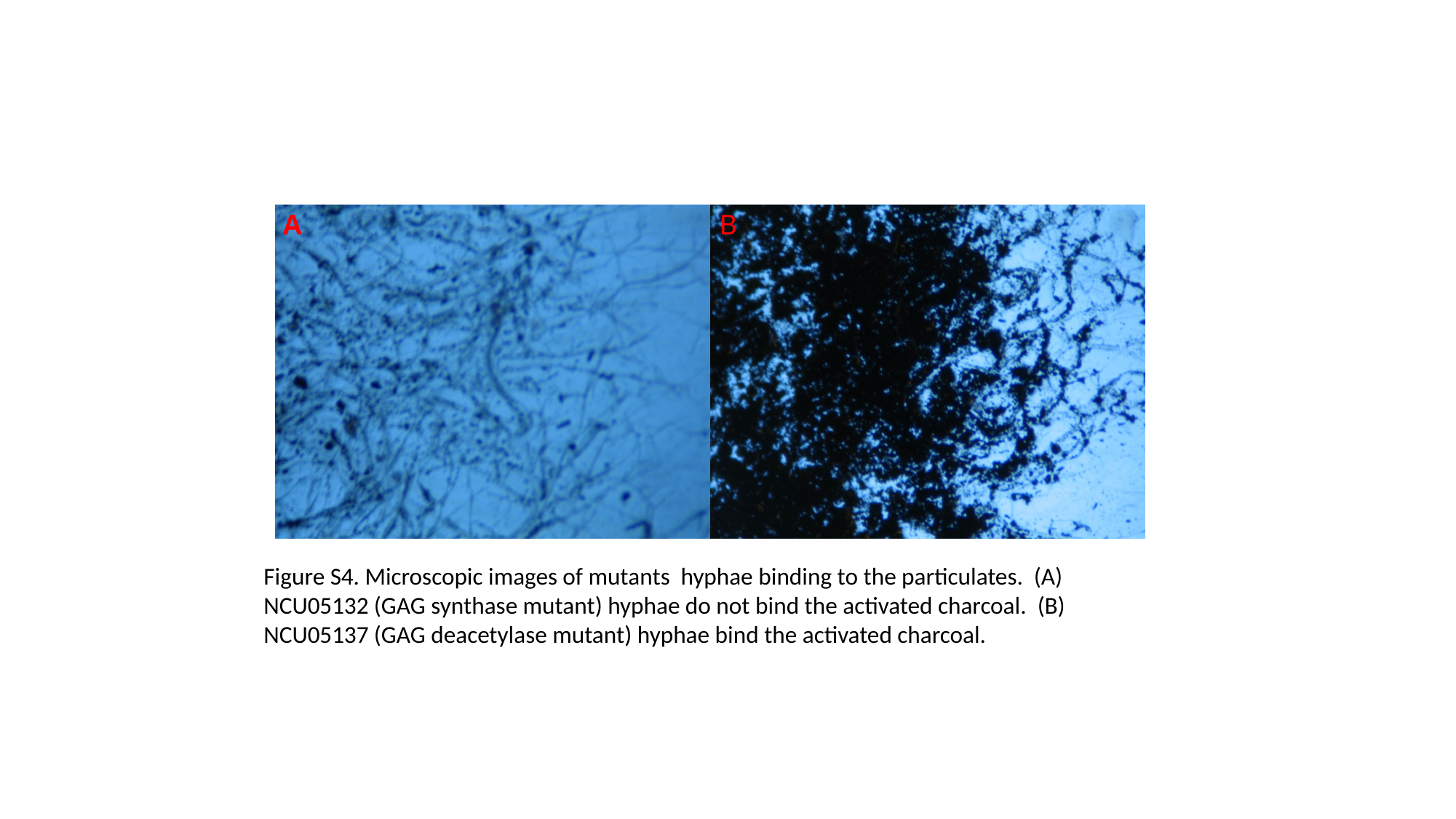

A
B

## Slide 5
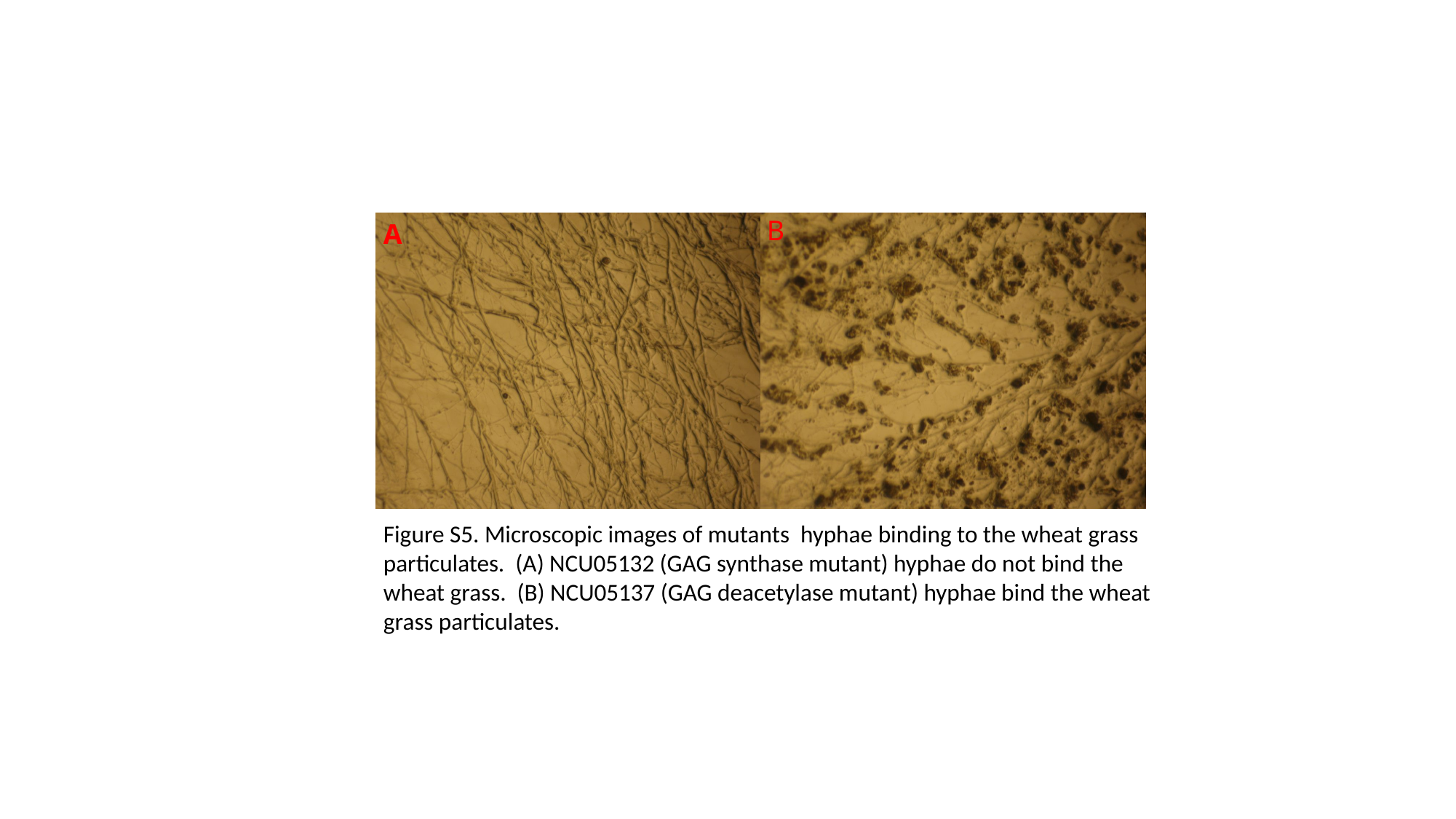

B
A
